# The Role of AFB1, OTA, TCNs, and Patulin in Forensic Sciences: Applications in Autopsy, Criminal Investigations, and Public Health Prevention

**DOI:** 10.3390/toxins16120514

**Published:** 2024-11-28

**Authors:** Matteo Antonio Sacco, Saverio Gualtieri, Alessandro Pasquale Tarallo, Maria Cristina Verrina, Angela Carbone, Wandamaria Mazzuca, Santo Gratteri, Isabella Aquila

**Affiliations:** Institute of Legal Medicine, Department of Medical and Surgical Sciences, “Magna Graecia” University, 88100 Catanzaro, Italy; matteoantoniosacco@gmail.com (M.A.S.); saveriogualtieri@icloud.com (S.G.); alessandropasquale.tarallo@studenti.unicz.it (A.P.T.); mariacristina.verrina@studenti.unicz.it (M.C.V.); angela.carbone1@studenti.unicz.it (A.C.); wandamaria.mazzuca@studenti.unicz.it (W.M.); gratteri@unicz.it (S.G.)

**Keywords:** AFB1, OTA, trichothecenes, patulin, autopsy, forensic science, crime, public health

## Abstract

Mycotoxins, specifically aflatoxin B1 (AFB1), ochratoxin A (OTA), trichothecenes (TCNs), and patulin, are a group of secondary metabolites that can contaminate food, leading to severe health implications for humans. Their detection and analysis within forensic toxicology are crucial, particularly as they can be implicated in cases of poisoning, foodborne illnesses, or lethal chronic exposure. However, little is known about the application that mycotoxins could have in forensic investigations and especially about the possibility of extracting and quantifying these molecules on tissues or post-mortem fluids collected at autopsy. We propose a review of the scientific literature on autopsy case studies in which the presence of mycotoxins on cadavers in cases of acute and chronic exposure has been investigated and identified. This review demonstrates how the analysis of mycotoxins on cadavers could be fundamental in the study of mushroom poisonings or even in the investigation of the chronic effects of mycotoxins on the human organism, by virtue of the known carcinogenic and mutagenic effects of many of them. This paper aims to explore the multifaceted role of mycotoxins within forensic sciences, focusing on their detection methods, implications in criminal contexts, and their potential as forensic evidence, thereby underscoring the critical importance they could assume in post-mortem toxicology, public health prevention, and forensic investigations.

## 1. Introduction

Mycotoxins are toxic substances produced by certain fungi, including *Aspergillus*, *Penicillium* and *Fusarium* species. Among them, aflatoxin B1 (AFB1), ochratoxin A (OTA), trichothecenes (TCNs), and patulin represent the most significant concerns for public health due to their severe toxic effects and potential relevance in forensic sciences. Aflatoxins are among the most potent mycotoxins affecting human health, known for their diverse and severe toxic effects. Due to fungal contamination that occurs both before and after harvest, aflatoxins are frequently found in foods such as rice, flour, corn, dried fruit, nuts, maize, rice, figs, spices, raw vegetable oils, and cocoa beans. Aflatoxins, due to their high fat solubility, are rapidly absorbed through the gastrointestinal tract and spread through the bloodstream. These toxins are produced by *Aspergillus*, a fungus particularly prevalent in hot, humid climates.

From a toxicity perspective, aflatoxin B1, specifically, is implicated as a carcinogen in human hepatocellular carcinoma (HCC), which is the second leading cause of cancer death worldwide. When ingested, inhaled, or absorbed through the skin, aflatoxins exhibit carcinogenic, hepatotoxic, teratogenic, and mutagenic effects, posing significant health risks even at low exposure levels [1]. Aflatoxin B1 (AFB1) is particularly notorious, being the most toxic and strongly associated with various health problems, including liver cancer [2]. Acute poisoning from high doses of aflatoxins, known as aflatoxicosis, can be life-threatening due to severe liver damage [3]. Furthermore, chronic exposure to these toxins, especially in conjunction with chronic hepatitis B virus infection, enhances their hepatocarcinogenic potential, leading to episodic cases of aflatoxicosis [4]. The impact of aflatoxins on human health is complex and can be influenced by several factors, such as age, sex, weight, diet, and exposure to infectious agents [1].

In a recent review, Pożarska et al. described numerous toxic effects related to AFB1, including myocardial cell apoptosis, lipid peroxidation, lung tumorigenesis, liver damage with the development of hepatocellular carcinoma, and neurodegeneration [5]. From a molecular perspective, the authors reported correlations between AFB1 effects and the liver enzymes MMP1 and MMP7, which may provide key insights into AFB1-induced liver lesions. Additionally, AFB1 ingestion induces significant changes in the COX-2 enzyme, leading to cellular inflammation, pyroptosis, steatosis, and mitophagy. Other effects of AFB1 include fertility impairment, as AFB1 can decrease LH levels and increase levels of progesterone, FSH, and hepatic alpha-fetoprotein (AFP). These effects can cause significant fetal developmental damage, including hydrocephalus, microphthalmia, and alterations in liver development.

Ochratoxins, another class of potent mycotoxins, are primarily produced by several *Aspergillus* and *Penicillium* species, posing substantial health risks due to their widespread presence in food and feed [6]. Ochratoxin A (OTA) is the most studied and concerning among them, with evidence linking it to several severe health issues. For instance, OTA exposure has been associated with Balkan endemic nephropathy (BEN) and chronic interstitial nephropathy (CIN) [7]. The toxicological profile of ochratoxins reveals carcinogenic, nephrotoxic, hepatotoxic, estrogenic, neurotoxic, and immunosuppressive effects [8]. This wide range of toxic effects makes ochratoxins particularly dangerous, as they can often remain undetectable in food products, posing a hidden threat to human health [8]. Another review highlighted how the liver aids in OTA elimination through “oatp carrier cells” and subsequent excretion by the kidneys via the “pAH transporter”. The bioaccumulation of OTA and its metabolites contributes to nephrotoxicity, as seen in BEN in humans and malignant tumors in pigs. The prolonged serum half-life of OTA contributes to its bioaccumulation and tumor promotion. Recent research also demonstrated OTA’s damaging cellular effects via activation of p38, JNK, and ERK, with potential correlations to neurodegenerative diseases like Alzheimer’s [9]. In recent work, Arce-Lopez et al. also found higher OTA levels in individuals with neurodegenerative diseases, such as Parkinson’s and Alzheimer’s, further indicating its neurotoxic effects [10].

TCNs represent a diverse group of mycotoxins produced by various fungal species, including Fusarium, Myrothecium, and Stachybotrys, and are a significant concern due to their toxicity to humans and other organisms [11]. The main trichothecenes include deoxynivalenol (DON), nivalenol (NIV), T-2 toxin, HT-2 toxin, and diacetoxyscirpenol (DAS), all of which exhibit potent toxic effects [12]. Exposure to trichothecenes has been linked to acute poisoning outbreaks affecting large populations [12]. The most notable toxic effect is alimentary toxic aleukia (ATA), reported from 1932 to 1947 due to overwintered grain consumption, with a high mortality rate (about 60%). Recent research has also linked the T-2 toxin to Kashin–Beck disease (KBD), a chronic joint disease observed in Eastern Siberia, China, and Korea. A study by Ning et al. recently evaluated HT-2 toxin and T2-terol levels in the urine of KBD patients compared to controls [13,14]. These toxins are harmful to various biological systems, including mammals, birds, fish, and plants, making their presence in the environment a considerable health risk [15]. The extensive toxicity of trichothecenes underscores the importance of monitoring and managing their presence in food and feed to safeguard human health.

Patulin exposure can lead to a wide range of acute and chronic health effects in both humans and animals. Acute toxicity is often characterized by symptoms such as nausea, vomiting, and diarrhea, which can be attributed to the immediate impact of patulin on gastrointestinal health [16]. More severe acute cases may result in ulceration, agitation, convulsions, edema, and even DNA damage, showcasing the mycotoxin’s potential to cause significant physiological distress [17]. On the other hand, chronic exposure to patulin, typically through low-dose consumption over an extended period, has been linked to life-threatening conditions such as immunotoxicity and potential carcinogenic effects [18]. Such chronic health implications underscore the critical need for understanding and managing patulin exposure to protect both human and animal health.

For these reasons, international regulatory bodies have established maximum permissible levels of mycotoxins in food and feed. Recent scientific literature reflects growing interest in mycotoxin analysis through the development of numerous studies focused on extraction and analytical methods, as well as in vitro and in vivo models. However, despite a substantial number of studies on the direct toxicology of these molecules, little is known about the impact this topic may have on forensic science. In particular, to date, the analysis of these toxins in humans, especially in cadavers, is poorly understood. This work aims to evaluate the current medico-legal implications of mycotoxin studies and the contribution that forensic sciences and cadaver analyses can offer in this field. To this end, we propose a review of the scientific literature on the effects of mycotoxins in cadavers, as well as methods for their extraction from autopsy fluids and tissues. Furthermore, research on other forensic applications of mycotoxins has been conducted.

## 2. Post-Mortem Evidence of Deaths due to Mycotoxins and Crime Scene Applications

This literature review reveals that limited research has explored this topic from a forensic medical perspective. However, we have outlined two main aspects related to the study of mycotoxins in which the contribution of forensic sciences can be decisive. These topics include the following: (1) post-mortem autopsy and toxicological investigation of organs and biological fluids in cases of suspected acute and/or chronic exposure with the development of specific protocols for the extraction of toxins from cadavers; (2) analysis of mycotoxins at the crime scene via forensic mycology.

### 2.1. Autopsy Evidence in Mycotoxin Analysis

#### 2.1.1. Aflatoxin Poisoning

We highlight that, although reported cases in the literature are few, autopsy can serve as a crucial investigative tool in cases of suspected mycotoxicosis, whether single or multiple, providing significant forensic insights. In cases of suspected poisoning, an accurate collection of circumstantial data is paramount. First, it is necessary to evaluate, through testimonial evidence, how the death occurred and whether there were witnesses present at the time. Information should also be gathered about the deceased’s occupation and related dietary habits. Investigators should then assess whether any identifiable signs at the scene suggest the ingestion of specific contaminants or potential contact with fungi.

In suspected cases of mycotoxicosis, the autopsy must include a thorough external and internal examination to identify signs consistent with mycotoxicosis.

Aflatoxins primarily target the liver, causing either acute aflatoxicosis, which can result in rapid liver damage and possible death, or chronic aflatoxicosis, which is associated with liver cancer, immune suppression, and other insidious diseases. Similar debilitating effects are observed in animals, with symptoms such as reduced growth rates, decreased milk production, and jaundice. Initially, individuals exposed to aflatoxins may experience nonspecific symptoms such as nausea, vomiting, and abdominal pain [19]. As exposure progresses, more severe symptoms can develop, including jaundice, a marker of liver damage, and in acute cases, fulminant hepatic failure [20]. In a retrospective study, Dereszynski et al. analyzed 72 cases of acute aflatoxin poisoning in dogs by examining signs and symptoms, histopathological findings, and the effects of treatment on survival [21]. Reported symptoms included anorexia, vomiting, diarrhea with melena and hematochezia, acute hepatitis, and hemorrhagic diathesis. Histological analysis revealed hepatocellular lipid vacuolation and fibroplasia with marked fibrosis [22].

During the external examination, the forensic pathologist should observe any signs of vomiting and diarrhea and collect information on the presence of symptoms related to abdominal pain before death. Additionally, signs of jaundice should be assessed by examining the sclerae and skin.

Next, during the autopsy, when opening the abdomen, the pathologist should evaluate any intra-abdominal effusions, quantify their volume, and describe their morphological characteristics. The liver should be thoroughly examined, noting its size, weight, consistency, and morphology, and any anatomical alterations should be documented. This examination should include anatomical sectioning to analyze the intrahepatic parenchyma and assess hepatic vascularization.

**Figure 1:**
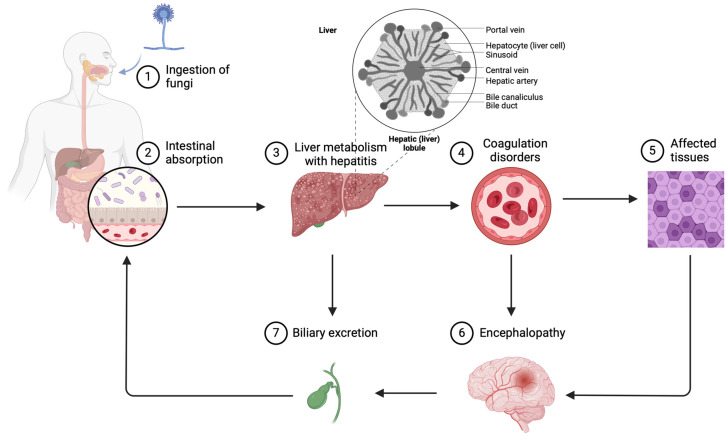
Effects due to poisoning in case of aflatoxicosis (created with Biorender.com, accessed on 1 October 2024).

Since aflatoxicosis is associated with fulminant hepatitis, it is essential to sample liver tissue, fixing it in 10% formalin for processing and embedding. Pathological and microscopic analyses should then be conducted to evaluate signs of acute hepatitis, including lobular pattern, degree of steatosis and fibrosis, and evidence of hepatic cytolysis or necrosis. Additionally, as aflatoxicosis is linked to hemorrhagic diathesis, it is necessary to check for signs of both external and internal hemorrhage (Figure 1).

This investigation should also be extended to the brain, considering the potential for acute encephalopathy. Macroscopic examination of the brain should include measurements of weight and dimensions, followed by a gross analysis to check for cerebral edema or hemorrhages. These areas should be sectioned and sampled for histological analysis to evaluate signs of neurological cytotoxicity.

In cases of acute intoxication, examination of the stomach is also essential to assess gastric contents, which should be sampled for toxicological analysis.

Chronic exposure to aflatoxins presents unique challenges, resulting in long-term health effects, such as cancer—particularly hepatocellular carcinoma—and immune system suppression [23]. In pets, symptoms of aflatoxin poisoning, including lethargy, loss of appetite, and visible jaundice, resemble those observed in humans, highlighting the toxin’s broad impact across species [24]. In cases of chronic exposure, it is therefore essential to assess for hepatic carcinoma. These lesions typically present as firm, whitish nodular areas in the liver parenchyma, requiring incision and sampling for histopathological investigation. In instances of immune suppression, it is also advisable to check for signs of pulmonary or systemic superinfection, although such cases of aflatoxicosis are generally accompanied by concurrent hepatic symptoms.

Aflatoxin toxicity levels vary according to several factors, influencing the severity and progression of poisoning. The route of exposure, with ingestion and inhalation being the main pathways for aflatoxins to affect humans and animals, plays a significant role. Dose and duration are also critical, with acute aflatoxicosis resulting from large doses over short periods, while chronic conditions emerge from prolonged, low-level exposure.

#### 2.1.2. Ochratoxin Poisoning

Ochratoxin exerts a profound biological impact on various organs and systems, particularly on the kidneys, where it displays nephrotoxic effects [25]. It is implicated in renal disease pathogenesis, notably in conditions such as porcine and poultry nephropathy. Once in the bloodstream, ochratoxin disrupts cellular energy production by impairing mitochondrial function, an early sign of toxicity [26]. Due to ochratoxin’s dose-dependent toxicity, its severity increases with higher exposure levels, potentially leading to cellular apoptosis, further compromising organ and systemic health. Symptoms and signs of ochratoxin poisoning range from acute to chronic, affecting multiple body systems. Initial symptoms, like gastrointestinal disturbances such as nausea, may precede more severe effects [27]. Mitochondrial dysfunction—marked by reduced ATP production and protein synthesis—is an early indicator of ochratoxin toxicity, leading to fatigue and metabolic imbalances. As ochratoxin’s effects progress, more pronounced symptoms, especially in the liver and kidneys, may emerge, resulting in organ damage. Recognizing these early signs is crucial for timely intervention to prevent long-term health issues associated with ochratoxin poisoning.

In cases of ochratoxin toxicity, it is essential to assess for signs of both hepatotoxicity and acute nephrotoxicity. This includes morphological analysis of kidney size and weight, followed by sectioning. The forensic pathologist should examine the cortex and medulla macroscopically and look for signs of acute nephrotoxicity during histological evaluation. Di Paolo et al. documented a case of acute renal failure due to inhalation of ochratoxin from Aspergillus ochraceus [28], noting tubulonecrosis indicative of nephrological damage on histological examination.

Acute trichothecene poisoning presents with rapidly progressing symptoms, beginning with gastrointestinal distress, such as vomiting, and a marked loss of appetite—potentially due to disruption of appetite regulation by enteric microorganisms. Skin blistering may also occur, indicating the toxin’s systemic impact on multiple organs. In severe cases, exposure can lead to significant weight loss and debilitation. At autopsy, it is therefore essential to assess nutritional status, weight, and thoroughly examine the gastrointestinal tract, including the stomach, duodenum, ileum, and colon, for signs of hemorrhage.

#### 2.1.3. Trichothecenes Poisoning

Trichothecene poisoning manifests through a spectrum of acute and chronic symptoms that vary depending on the level and duration of exposure. Acute symptoms are often severe and can include vomiting, diarrhea, and anorexia, as observed in both humans and experimental animal models [12,13,14,15]. These symptoms are indicative of the toxin’s rapid irritation to the skin and intestinal mucosa [12,13,14,15]. In more severe cases, acute exposure can lead to hemorrhages, stomach necrosis, and dermatitis, which are critical indicators of the toxin’s damaging impact on the body’s cellular structures. Chronic exposure, on the other hand, presents as growth retardation, immunotoxicity, and persistent anorexia, reflecting the prolonged interference of trichothecenes with normal bodily functions [12,13,14,15]. This variability in symptoms underscores the necessity for prompt diagnosis and intervention to mitigate the adverse health effects of trichothecene exposure.

#### 2.1.4. Patulin Toxicity

Patulin intoxication also produces toxicity symptoms, with acute exposure manifesting as gastrointestinal issues such as nausea, vomiting, ulcers, and intestinal inflammation.

Although patulin–cysteine adducts are less toxic, they still pose a risk with larger doses or prolonged exposure [29]. The long-term health effects of patulin exposure are particularly concerning, with conditions such as teratogenicity, carcinogenicity, and mutagenicity documented in studies involving excessive intake [30]. Patulin’s reactivity with thiol groups within cells contributes to its toxicity, impacting various biological systems and potentially leading to long-term organ damage. These health risks underscore the importance of continued research and regulatory efforts to mitigate patulin exposure.

### 2.2. Analysis of Mycotoxins in Cadaveric Biological Fluids or Tissues

The forensic pathology literature highlights the scarcity of studies on post-mortem mycotoxin identification in human cadavers. Qureshi et al. observed a high prevalence of AFB1 in autopsied children’s brains, associating it with cytotoxic effects on the microvascular endothelial barrier. They found that a 24 h exposure to AFB1 killed over 85% of human brain microvascular endothelial cells (HBMEC) and 22% of human umbilical vein endothelial cells (HUVEC) at a concentration of 32 nM, suggesting aflatoxins’ ability to cross the blood–brain barrier and cause damage [31]. In studies from Nigeria, aflatoxins were found in multiple organs, particularly in children with kwashiorkor and other diseases, revealing no concentration differences between groups [32,33,34]. Aflatoxins in brain tissue were prevalent, likely due to their lipophilic nature, leading to accumulation [33]. Research in Ghana linked aflatoxin B1 to kwashiorkor in liver tissue samples from autopsied children, supporting a potential etiological role in the disease [35]. Similar studies confirmed aflatoxin presence in lungs and liver of kwashiorkor-affected children [36,37,38,39]. In Japan, at Juntendo University School of Medicine, aflatoxins B1, B2, and M1 were isolated from lung and skin lesions in a leukemia patient with Aspergillus flavus infection, demonstrating aflatoxins’ localized impact and systemic immune implications [40].

Grey et al. reported the case of a 31-year-old exposed to toxic spore levels (up to 29,000 spores/m^3^) in his home, who developed pancytopenia and severe respiratory failure. The autopsy revealed trichothecenes (2.5–3.25 ppb) and aflatoxins (6.0 ppb) in the liver and lungs, while the brain tested positive for aflatoxins (5.5 ppb), ochratoxins (170 ppb), and trichothecenes (2.05 ppb) [41]. In a recent study by Castell et al., the presence of enniatins (A, A1, B, and B1) and beauvericin was evaluated in multiple organs, including brain, lung, kidney, fat, liver, and heart. The researchers used solid–liquid extraction (SLE) followed by dispersive liquid–liquid microextraction (DLLME) [42]. For SLE, ultrapure water, acetonitrile, and sodium chloride were used, and for DLLME, chloroform extraction was performed. Analysis through liquid chromatography coupled with tandem mass spectrometry (HPLC-MS/MS) revealed significant bioaccumulation of mycotoxins in humans, with Enniatin B (ENNB) detected in 100% of liver (7 ± 13 ng/g) and fat samples (0.2 ± 0.8 ng/g), while low concentrations were found in the lungs and heart. Additional cases include the identification of satratoxin H in a fatal mushroom-poisoning case involving Podostroma cornu-damae, where the authors used LC-QTOF-MS/MS to detect various mycotoxins in blood and stomach samples [43].

### 2.3. Impact of Mycotoxins on the Crime Scene

To date, no studies aimed at the application of mycotoxins at crime scenes have been found in the literature. Specifically, the only studies identified thus far relate to the analysis of forensic mycology, focusing on the use of fungi for forensic purposes through macroscopic study.

Spychala et al. recently described some potential applications of forensic mycology in a literature review [44]. The authors highlighted that, despite numerous studies on forensic bacterial microbiology, very few studies have focused on fungi. Potential applications include geolocating crime scenes and estimating the post-mortem interval. Povilauskas et al. described the case of a man reported missing in an area of Buenos Aires, Argentina. The authors noted that the victim was found after 25 days, with a high quantity of *Bipolaris cynodontis* present, which allowed for geographical reconstruction of the crime scene [45]. Forensic mycologists meticulously collected fungal spores from exposed areas of the victim’s body, which had developed extensive fungal growth over time. These samples were analyzed to determine unique spore profiles that could potentially correlate with environmental conditions. The analysis underscored the capability of fungal spores to serve as trace evidence, providing insights into the timing and nature of the victim’s exposure to environmental elements.

The authors emphasized a correlation between fungal development and cadaveric decomposition. As a body decomposes, it creates an environment conducive to fungal colonization, which varies according to environmental conditions and stages of decomposition. These growth patterns can be influenced by factors such as temperature, humidity, and the presence of other decomposing organisms. For instance, certain fungi are known to proliferate during extended post-mortem periods or in environments that inhibit bacterial and scavenger activity, offering unique clues about the timing and duration of decomposition.

Gemmellaro et al. demonstrated how DNA metabarcoding studies can aid in identifying and quantifying the microbial community and in studying fungal succession. The authors evaluated the decomposition of six pigs and noted an increase in the proportion of fungi during decomposition, especially in more advanced stages of putrefaction with certain species unique to the process [46].

Recently, Tranchida et al. reported a homicide case in a confined space where high humidity allowed fungal growth [47]. The study of fungal cultures from furniture enabled the determination of the time of death. The primary species identified at the scene were *Mucor plumbeus* Bonord, *Penicillium brevicompactum* Dierckx, and *P. citrinum* Thom. [47].

Other studies have also highlighted the potential of fungi for forensic purposes, with various applications including suspect identification, cause-of-death analysis, evaluation of the primary crime scene, putrefaction, and forensic archaeology [47,48,49,50,51,52,53]. One significant limitation in using fungi for crime scene analysis is the risk of contamination, which can greatly affect the integrity of results. Contamination can occur when fungal samples are inadvertently introduced from external sources, leading to erroneous data that may misrepresent actual crime scene conditions. This challenge is particularly critical in complex environments where multiple fungal species coexist, making it difficult to discern which fungi are relevant to the crime. In forensic mycology, extraneous fungal DNA can obscure evidence, complicating analysis and interpretation. To mitigate these issues, strict protocols are essential to ensure sample collection and preservation in a way that minimizes contamination. This involves using sterile equipment, proper labeling, and maintaining a controlled environment during analysis.

Distinguishing between human and environmental fungal traces presents another challenge in forensic analysis. The ubiquity of fungi in the environment means they are naturally present at most crime scenes, complicating the task of identifying fungi directly associated with human activity. Forensic mycology must, therefore, employ sophisticated techniques to differentiate these sources. This is crucial, as environmental fungi could be mistaken for evidence linked to the crime, potentially leading to incorrect conclusions [47,48,49,50,51,52,53].

## 3. Medico-Legal Investigations Using Mycotoxins

Mycotoxins, essentially secondary metabolites produced by microfungi, play a significant role in forensic science due to their potential to cause diseases and even death in humans and other animals. These toxic compounds are synthesized by a variety of filamentous fungi, notably including *Aspergillus*, *Penicillium*, and *Fusarium* species, which are notorious for their serious health implications on exposed populations.

In forensic toxicology, the detection and analysis of mycotoxins are critical for elucidating cases of poisoning and death where mycotoxins are suspected causative agents. The role of mycotoxins in forensic toxicology extends beyond identification; it involves the complex process of evaluating their presence across various matrices, including food, biological tissues, and environmental samples. Since mycotoxins have the potential to cause not only chronic but also acute effects in humans, with lethal consequences such as intoxication and death, it is necessary to consider which signs should be evaluated on a corpse, which toxins are most toxic, and above all, the methods for assessing these effects. Toxins can be extracted, isolated, and quantified. The literature review shows how issues related to mycotoxin exposure in forensic settings can be varied.

### 3.1. Protocol of Autopsy and Post-Mortem Investigations

**Figure 2:**
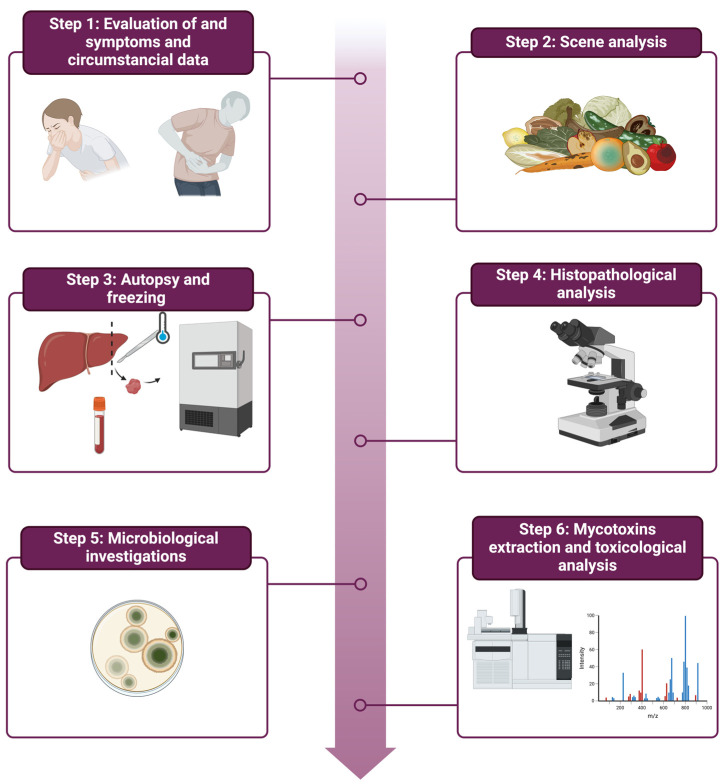
Protocol for post-mortem investigations in suspects of mycotoxin related death (created with Biorender.com, accessed on 1 October 2024).

As described in the results, an accurate evaluation of the available circumstantial data and autopsy is essential in order to identify signs suggestive of mycotoxicosis. To this end, we propose the following protocol: (1) collection of testimonial information about the symptoms reported by the subject before death, work performed, and eating habits; (2) analysis of the death scene with evaluation of traces attributable to food and subsequent seizure for investigations; (3) evaluation of signs (such as traces of vomiting and hematemesis) during external cadaveric examination; (4) analysis of the organs with particular reference to the liver and gastrointestinal tract; (5) opening of the stomach with collection of gastric contents for toxicological investigations; (6) sampling of tissues with formalin fixation for the evaluation of histopathological signs; (7) microscopic search of lung tissue for fungi in case of inhalation of contaminants; (8) peripheral blood sampling with post-mortem microbiological analysis of fungi; (9) preservation of tissue samples of liver, kidney, brain, and heart at −80 °C for the extraction investigations of the investigated mycotoxins (Figure 2).

### 3.2. Extraction of Mycotoxins in Autopsy

Detecting mycotoxins in post-mortem analysis involves laboratory techniques that present unique challenges. Thin-layer chromatography (TLC), gas chromatography (GC), and high-performance liquid chromatography (HPLC) coupled with mass spectrometry (MS) are some of the primary methods used to identify and quantify these compounds [35]. Each technique offers distinct advantages, such as TLC’s simplicity and cost-effectiveness, GC’s high resolution, and HPLC-MS’s sensitivity and specificity. However, the choice of method often depends on the specific mycotoxin being investigated and the available laboratory resources. Innovations in enzyme-linked immunosorbent assay (ELISA), lateral flow assays (LFAs), and biosensors have further improved the accuracy and efficiency of mycotoxin detection in autopsy samples [4]. These methods not only provide rapid results but also require minimal sample preparation, making them ideal for forensic applications.

Several case studies have documented fatalities linked to mycotoxin exposure. For instance, mycotoxins have been detected in body fluids, biopsies, and autopsy specimens of individuals exposed to molds in water-damaged indoor environments [30]. One notable case involved the detection of fungal DNA in autopsy specimens using real-time polymerase chain reaction (RT-PCR), alongside mycotoxins identified viathe ELISA method [41]. Such findings highlight the importance of thorough toxicological analyses in post-mortem investigations to ascertain the presence and impact of mycotoxins. Moreover, these case studies underscore the diverse sources of mycotoxin exposure, ranging from contaminated food to environmental molds, necessitating a multidisciplinary approach to public health and safety.

Identifying mycotoxin poisoning during autopsies presents several challenges, primarily due to the complexity and variability of these toxins. One major obstacle is the degradation of mycotoxins over time, which can complicate the detection process if samples are not collected and analyzed promptly. Additionally, the need for well-developed analytical methods remains a significant issue, as the accuracy and reliability of mycotoxin detection heavily depend on the techniques employed. Traditional methods, like chromatographic and immunochemical techniques, are often supplemented with newer approaches such as biosensors and optical methods to enhance detection capabilities [54,55]. Despite these advancements, providing reliable data to support the risk assessment of foodborne mycotoxins continues to be a critical challenge in forensic toxicology [56].

### 3.3. The Link Between Mycotoxins and Kwashiorkor

Epidemiological studies have provided substantial evidence linking mycotoxin exposure to the occurrence of kwashiorkor. Numerous investigations have highlighted the prevalence of aflatoxins in regions where kwashiorkor is endemic [57]. These studies indicate that aflatoxins are found in higher concentrations in the biological samples of individuals diagnosed with kwashiorkor compared to those with other forms of malnutrition, such as marasmus or marasmic-kwashiorkor [57]. The geographical correlation between mycotoxin contamination in food supplies and the incidence of kwashiorkor further supports this association. Notably, dietary staples in many developing countries, such as maize and peanuts, are particularly susceptible to mycotoxin contamination, creating a significant public health risk [58].

The mechanisms through which mycotoxins induce protein deficiency and contribute to kwashiorkor are multifaceted. Some mycotoxins, such as aflatoxins and fumonisins, are known to interfere with protein synthesis within the body, thereby exacerbating protein malnutrition [59]. When these mycotoxins are ingested, they can disrupt various metabolic pathways, leading to impaired liver function and a reduction in the synthesis of essential proteins. This disruption is critical in the context of kwashiorkor, where protein deficiency is a hallmark of the disease. Additionally, mycotoxins can compromise the immune system, making individuals more susceptible to infections, which can further deplete protein reserves [60].

Multiple case studies have highlighted the connection between mycotoxin exposure and the development of kwashiorkor. For instance, research by Hendrickse et al. explored the link between aflatoxin exposure and kwashiorkor in African children, revealing that those with kwashiorkor had significantly higher levels of aflatoxins in their serum than healthy controls [38]. Another study emphasized the presence of aflatoxins in various organs of affected individuals, suggesting a direct correlation between aflatoxin burden and the severity of kwashiorkor symptoms [61]. These case studies underscore the critical need for further research to elucidate the precise mechanisms by which mycotoxins contribute to kwashiorkor and to develop effective strategies for mitigating this public health issue.

### 3.4. Crimes Related to Mycotoxins

Throughout history, mycotoxins have played a disturbing role in various incidents, some of which have had devastating consequences. One notable historical incident is the Aflatoxicosis outbreak in Kenya in 2004, where over 100 people died, and hundreds more suffered from severe health complications [62]. This tragedy underscored the lethal potential of mycotoxins when they contaminate food supplies, a risk that has been present since humans began cultivating and storing crops [63].

In modern times, instances of mycotoxin use in crimes have become subjects of intense investigation. The complexity of investigating such cases lies in the diverse nature of mycotoxins, with more than 300 identified types, each with varying effects and occurrences in different foodstuffs. Establishing guidelines to control mycotoxins remains a formidable challenge despite legal regulations implemented by many countries for food quality assurance. One of the primary hurdles in forensic investigations is the complexity of the matrix in which mycotoxins are found, which can complicate the detection and analysis processes. Moreover, the concentration of these toxins is often below detectable levels, necessitating advanced extraction and detection technologies [64].

### 3.5. Health Impacts of Mycotoxin Exposure

Acute toxicity due to mycotoxin exposure can lead to immediate and severe health effects. One of the most notable examples is acute aflatoxicosis, characterized by liver damage, jaundice, lethargy, and even death in severe cases [65]. This form of toxicity often arises from consuming contaminated food products, particularly in regions with inadequate food safety measures [66]. Symptoms can manifest rapidly and are often severe, requiring urgent medical attention.

Chronic diseases resulting from prolonged mycotoxin exposure can have debilitating effects on human health. Long-term exposure to mycotoxins such as aflatoxins is associated with a range of chronic conditions, including liver cancer, immune suppression, and other slow-progressing pathological conditions. These toxins can accumulate in the body over time, leading to persistent health issues that may be difficult to diagnose and treat. For instance, chronic exposure to aflatoxins, especially in conjunction with hepatitis B virus infection, significantly increases the risk of hepatocellular carcinoma [67]. The impact of chronic mycotoxin exposure is particularly pronounced in developing nations, where food safety standards may be less stringent, leading to higher rates of contamination.

The carcinogenic effects of mycotoxins are a major concern for public health. Several mycotoxins, including aflatoxins and ochratoxin A (OTA), have been classified as human carcinogens by the International Agency for Research on Cancer (IARC) [68]. These toxins can induce mutations and disrupt normal cellular processes, leading to cancer development over time. For example, aflatoxins are known to be hepatocarcinogenic, particularly when combined with chronic hepatitis B virus infection [68]. The presence of OTA in human serum and milk further underscores the widespread exposure and potential carcinogenic risk. Addressing these risks requires comprehensive strategies to monitor and control mycotoxin levels in food supplies, especially in vulnerable populations.

### 3.6. The Role of Mycotoxins on the Crime Scene

Forensic mycology, a specialized branch of forensic science, plays a crucial role in crime scene analysis through the identification of fungal species [41,42,43,44,45,46,47,48,49,50]. By analyzing the fungal biota present, forensic investigators can establish a connection between the crime scene and the defendant, aiding in the reconstruction of events leading up to the crime. This process involves the meticulous examination of fungal spores, hyphae, and other fungal structures found at the scene. These fungal elements can serve as trace evidence, thus providing critical information about the environment and conditions of the crime scene. For instance, specific fungal species may be indicative of certain geographical locations or environmental conditions, thereby narrowing down the possible origins of the crime scene.

Moreover, the presence of certain fungi can suggest the introduction of foreign material or the movement of objects within the scene, further aiding in the investigative process. Another significant application of forensic mycology is in determining the post-mortem interval (PMI). The growth patterns and succession of fungi on decomposing remains can offer valuable clues about the elapsed time since death [44,45,46,47,48,49,50,51,52,53]. Fungi colonize a corpse in predictable stages, and by identifying the species present and their developmental stages, forensic mycologists can estimate the PMI with a reasonable degree of accuracy.

No studies have yet examined the use of mycotoxins at crime scenes. However, since mycotoxins are a direct product of fungi, mycotoxins could also serve as valuable chemical markers in crime scene analysis. By analyzing the presence and concentration of mycotoxins, forensic experts could infer various details about the crime scene, such as the geographical origin of substances or the timeline of events. Additionally, DNA profiling techniques can be employed to link plant material containing mycotoxins to a specific location, further strengthening the connection between the suspect and the crime scene [44,45,46,47,48,49,50,51,52,53]. This multifaceted approach not only enriches the understanding of the crime but also strengthens the evidentiary value of mycotoxin analysis in forensic investigations.

Some case studies have demonstrated the critical role of mycotoxin evidence in solving complex forensic cases. For instance, the application of advanced methodologies such as DLLME HPLC–MS/MS has allowed forensic scientists to study and assess the incidence of emerging mycotoxins in human tissues, providing pivotal data in criminal cases, as reported in our review. To this end, we propose the application of forensic mycotoxicology, in a manner that overlaps with existing forensic botany and forensic bacterial microbiology, as further forensic biological evidence. For example, mycotoxin dosage could provide more detailed information on the scene (such as the soil) or on the degree of cadaveric decomposition by observing the chronological succession of fungi in the process of putrefaction.

### 3.7. Future Perspectives and Recommendations

In developed countries, there is widespread recognition of the necessity to impose limits on mycotoxin concentrations in food and feed, which has led to the establishment of stringent regulations. These regulations are often designed to safeguard public health by ensuring that the levels of mycotoxins in consumables do not exceed established safety thresholds, for instance, the European Commission’s Regulation (EC) No 1881/2006 sets maximum levels for certain contaminants in foodstuffs [69]. However, the effectiveness of these regulations can vary significantly across different regions. In many parts of the world, regulatory standards for mycotoxins in food may have little or no impact on actually reducing mycotoxin risk due to enforcement challenges and lack of resources. Therefore, to enhance the efficacy of these regulations, there needs to be a concerted effort to harmonize standards globally and ensure robust enforcement mechanisms.

**Figure 3:**
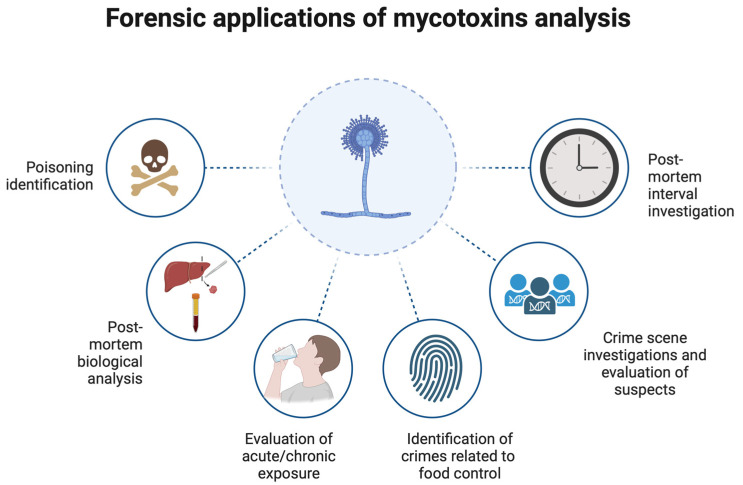
Medico-legal applications of mycotoxins (created with Biorender.com, accessed on 13 November 2024).

Autopsy and post-mortem investigations can offer a decisive contribution to controlling mycotoxin exposures in a wide range of contexts. Mycotoxicology could benefit from the reciprocal contributions of forensic sciences. This could include the evaluation of acute and chronic toxicity cases on cadavers, with isolation of toxins and quantitative assessment of dosage, potentially playing a key role in diagnosing mushroom poisoning cases or cases of chronic exposure to mycotoxins that might correlate with chronic or neoplastic diseases. Additionally, similar to forensic botany and forensic microbiology, mycotoxicology could be applied at crime scenes as a further source of biological evidence in investigations (Figure 3).

## 4. Conclusions

Mycotoxins, being potent toxic agents, can lead to severe health consequences, including acute poisoning and long-term health effects. The detection of mycotoxins in post-mortem biological samples is a crucial aspect of forensic toxicology, providing insights into human exposure and potential poisoning cases. Advanced detection methods have been developed to identify mycotoxins in various biological matrices such as plasma, serum, urine, and blood. These methods are essential for biomonitoring mycotoxin exposure in populations, which aids in understanding the prevalence and risk associated with these toxic compounds. Modern analytical techniques, including liquid chromatography coupled with mass spectrometry (LC-MS), have significantly enhanced the sensitivity and specificity of mycotoxin detection. By understanding the presence and concentration of mycotoxins in biological samples, forensic experts can better assess the impact of these toxins on human health and contribute valuable information to criminal investigations.

The role of mycotoxin analysis in criminal investigations extends beyond simple detection. Forensic analysis of mycotoxins could also potentially aid in identifying patterns of exposure in specific populations, helping to uncover potential criminal activities related to food safety violations. Case studies have underscored the forensic applications of mycotoxin analysis and mycology, demonstrating their utility in real-world scenarios which could be useful for justice.

## Data Availability

No new data were created or analyzed in this study. Data sharing is not applicable to this article.
